# Improving temporal accuracy of human metabolic chambers for dynamic metabolic studies

**DOI:** 10.1371/journal.pone.0193467

**Published:** 2018-04-24

**Authors:** Shanshan Chen, Erica Wohlers, Eric Ruud, Jon Moon, Bin Ni, Francesco S. Celi

**Affiliations:** 1 Department of Biostatistics, Virginia Commonwealth University, Richmond, Virginia, United States of America; 2 Department of Internal Medicine, Virginia Commonwealth University, Richmond, Virginia, United States of America; 3 MEI Research Ltd, Edina, Minnesota, United States of America; University of Alabama at Birmingham, UNITED STATES

## Abstract

Metabolic chambers are powerful tools for assessing human energy expenditure, providing flexibility and comfort for the subjects in a near free-living environment. However, the flexibility offered by the large living room size creates challenges in the assessment of dynamic human metabolic signals—such as those generated during high-intensity interval training and short-term involuntary physical activities—with sufficient temporal accuracy. Therefore, this paper presents methods to improve the temporal accuracy of metabolic chambers. The proposed methods include 1) adopting a shortest possible step size, here one minute, to compute the finite derivative terms for the metabolic rate calculation, and 2) applying a robust noise reduction method—total variation denoising—to minimize the large noise generated by the short derivative term whilst preserving the transient edges of the dynamic metabolic signals. Validated against 24-hour gas infusion tests, the proposed method reconstructs dynamic metabolic signals with the best temporal accuracy among state-of-the-art approaches, achieving a root mean square error of 0.27 kcal/min (18.8 J/s), while maintaining a low cumulative error in 24-hour total energy expenditure of less than 45 kcal/day (188280 J/day). When applied to a human exercise session, the proposed methods also show the best performance in terms of recovering the dynamics of exercise energy expenditure. Overall, the proposed methods improve the temporal resolution of the chamber system, enabling metabolic studies involving dynamic signals such as short interval exercises to carry out the metabolic chambers.

## 1 Introduction

Human metabolic chambers are essential tools for metabolic studies that require overnight stays, environmental control (e.g. ambient temperature, humidity, light and sound), and long duration resting [1]. Dietitians use them to study the effects of food and diet induced thermogenesis on human metabolism [2]. Endocrinologists and physiologists use them to study the effects of environmental changes and medical interventions on human metabolism [3]. Such studies often require long-term, continuous monitoring with environmental control to capture the response to intervention, and metabolic chambers are perfect tools for this.

While this near free-living environment provides comfort to the human subjects and the flexibility to enable various types of studies, it also presents challenges in measuring energy expenditure (EE) with high temporal resolution. In essence, the chamber system detects the subtle gas concentration changes in the chamber air caused by the respiration of the human subject housed in it, whereas the goal of the system is to detect the volume of O_2_ consumption (*VO*_2_) and the volume of CO_2_ production (*VCO*_2_) of the subject, and extrapolate energy expenditure (EE) and metabolic rate (MR, i.e. EE per unit time) from *VO*_2_ and *VCO*_2_ [4]. This creates an inverse system, where the inputs (i.e. *VO*_2_ and *VCO*_2_) to the system are the desired but hidden variables, and the outputs (O_2_ and CO_2_ concentration in the chamber air) are the direct measurements carrying little information of interest. This inverse problem (i.e. finding the input given the output of the system) is challenging in itself when the analytical transfer function of the system is unknown [5]. Moreover, the chamber system also functions as a low pass filter [6]. The large room can be considered as a big gas tank that dilutes subtle input signals (small amounts of *VO*_2_ and *VCO*_2_ generated by the subject), or equivalently, attenuates the high frequency portion of the signals, preventing the recovery of the input signals with sufficient temporal resolution.

Although this temporal resolution loss does not affect daily resting MR assessment in the typical metabolic studies designed to use the chamber, it can prevent the metabolic chamber from being a versatile tool for dynamic metabolic studies. For example, in sports medicine, where exercise is concerned, the time to reach a metabolic steady state and the recovery time to baseline of a subject are important metrics for assessing the subject’s physique. Moreover, traditional metabolic studies done in chambers usually impose sedentary activities on the subjects, which do not reflect real-world metabolic profiles, where physical activities are inevitable. Last but not least, a faster chamber response can shorten the waiting time when measuring resting energy expenditure as well.

This paper aims to improve the temporal accuracy of the chamber system in order to facilitate dynamic metabolic studies. To provide sufficient accuracy, step-by-step methods are used to estimate chamber volume, calibrate gas analyzers, and solve a site-specific problem. To improve temporal resolution, the paper proposes using a step size of one minute to compute the finite derivative terms, and applying a robust noise reduction method—total variation denoising—to minimize the large noise caused by the short derivative term.

The rest of the paper is organized as follows. Section 2 details the system design of the chamber system from which our experimental data were collected. Section 3 summarizes related work and the precursory approaches our proposed methods are based on. Section 4 proposes a noise reduction method to improve temporal resolution. Section 5 presents the results, showing improvement in both temporal resolution and accuracy using the proposed method on 24-hour infusion validation tests and a human exercise study. Section 6 summarizes the analysis and concludes the paper.

## 2 System design

**Fig 1:**
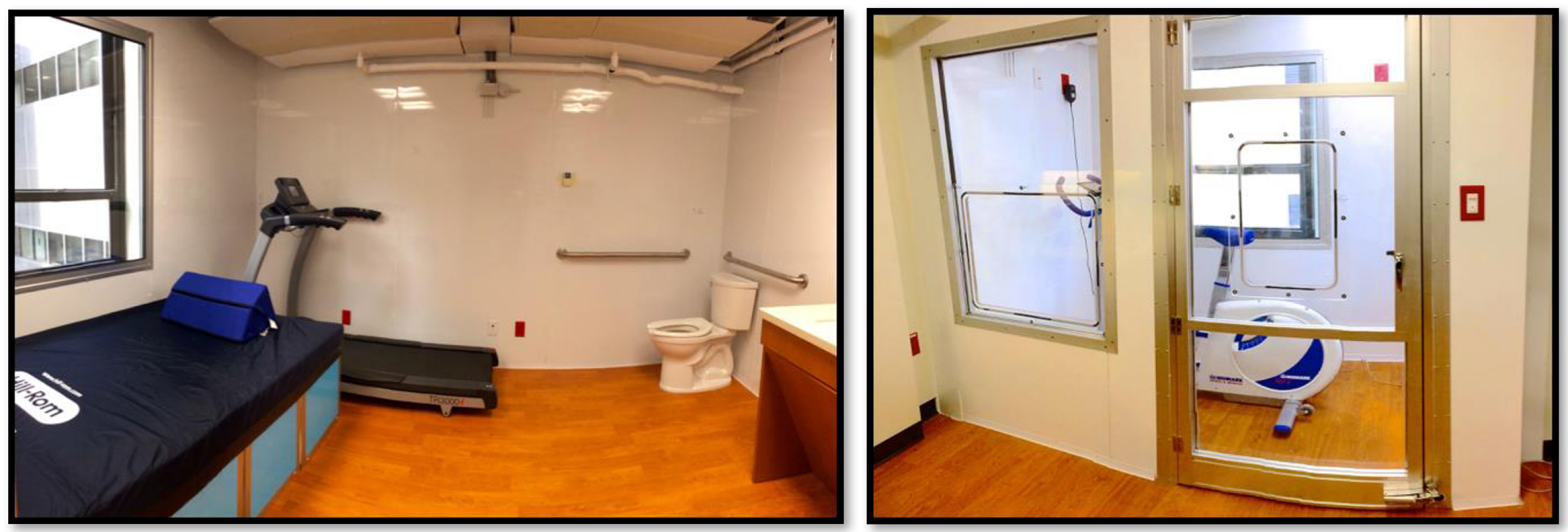
VCU metabolic chambers. Left: big chamber as a living room; right: small chamber (flex room) for single activities. Boxes can be added to the small chamber for resting energy expenditure studies.

The metabolic chambers described in this paper were constructed and integrated by MEI Research (Edina, MN), are located in the North Hospital on the medical campus of Virginia Commonwealth University (VCU, Richmond, VA). One chamber is the size of a living room (3m x 4m x 2.4m, “big chamber”) and can house a treadmill, a bike, a small desk, a toilet, and a wash basin at the same time. The other chamber is smaller (1.2 x 2.1 x 2.3 m, “small chamber”), with its volume further reduced by fitting in a set of bed boxes and a mattress, for a faster and more accurate response in energy expenditure readings. Both chambers are sealed and maintain positive pressure during data collection. Blood ports are also installed in the glass doors for in vivo blood sampling without the subject leaving the chamber. The chambers also have climate control, with an air conditioning (HVAC) system utilizing chilled water pipelines and electric heating coils, equipping the chambers for temperature-sensitive metabolic studies [3]. The settings of the two chambers are shown in Fig 1.

The big chamber has both “push” and “pull” modes for the fresh air to flow through. The small chamber has only the “push” capability, replenishing the room air by constantly flowing fresh air and passive ventilation. The fresh air for both chambers is directly sourced from the medical air system operated by the plant facility at VCU. The medical air system draws ambient air, filters, dries and compresses it, and sends the clean medical air to each hospital room via medical air pipelines. The inflow rates of the input medical air can be set from 20 liters per minute (l/min) to 100 l/min for both chambers, regulated by mass flow controllers (MFCs, manufactured by Porter Instrument, Hatfield, PA). Since the low pass filtering effect is proportional to the chamber volume, the small chamber’s temporal resolution will surpass the big chamber’s, given the same conditions and post-processing method. Therefore, in the rest of this paper, we will use the big chamber’s data to illustrate our method.

**Fig 2:**
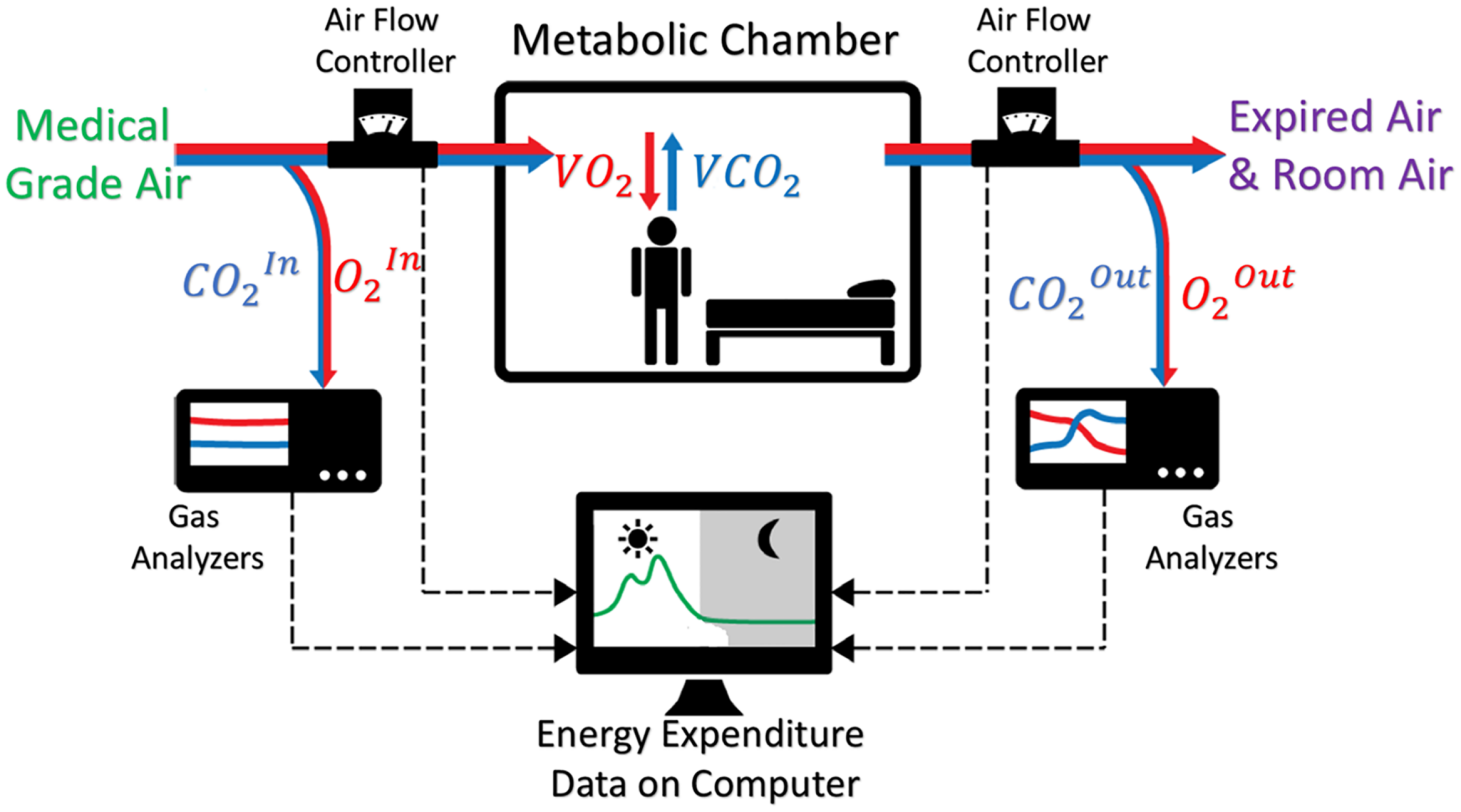
Concept diagram of the metabolic chamber. The chamber is configured as a push type of calorimeter with absolute gas analyzers measuring O_2_ and CO_2_ concentrations of both inflow air (medical grade air) and outflow air (expired air from human breath mixed with room air).

The concept of the chamber system is illustrated in Fig 2, revealing an inverse system where the inputs of the system are the desired variables (*VO*_2_ and *VCO*_2_). In general, the chamber system monitors critical variables such as inflow air rate, O_2_ concentration in inflow air ($O_{2}^{In}$) and room air ($O_{2}^{Out}$), CO_2_ concentration in inflow ($CO_{2}^{In}$) and room air ($CO_{2}^{Out}$), room temperature, room pressure, and room humidity. All these variables are logged by a computer in the control lab via integrated software developed in LabVIEW for post-processing in Matlab^®^.

To measure the changes in gas concentration levels in the chambers, the gas concentrations of the inflow air and the outflow air are constantly monitored at a sampling rate of once per minute. This is done by first drawing air at a rate of 2 l/min from the inflow air and outflow air, drying the air samples to a level below 1000ppm using a gas sample dryer (manufactured by Perma Pure LLC, Lakewood, NJ), and then measuring the air samples’ gas concentrations using gas analyzers (manufactured by Siemens, model: Ultramat/Oxymat 6). In order to maximize the resolution of the gas analyzers, the range of the O_2_ gas analyzers is set to 20% ∼ 21%, and the range of the CO_2_ gas analyzers is set to 0% ∼ 1%, which are the normal ranges of room air concentration with a human subject in it.

## 3 Related work

Three schools of approach were developed to solve the inverse problem mentioned in Section 1. The first school resorted to trend identification and exponential curve fitting with time sliding windows (approximately 20–30 minutes long) [7] [8]. Although it can recover steady-state MR signals with a long duration, it performs poorly during the dynamic phase due to the limitation of the long window size. The second school considered the chamber as a linear time-invariant system, and set up the inverse problem as a ill-conditioned deconvolution problem. Therefore, the inverse problem was transformed to identifying the transfer function of the system and solving the deconvolution with regularization [9] [10]. Using a theoretical transfer function, this method was able to achieve a 16-minute temporal resolution from a 16.6 m^3^ chamber with inflow rates of 50 −150 l/min. However, for dynamic MR signals during exercise, a much higher temporal resolution (e.g. 5 mins) is often desired. The third school modeled the instantaneous MR signal as a linear combination of recorded gas concentration data and its derivative [11] using the Z-Transform method, hence transforming the inverse problem to a direct problem [12] [13] [14] [15]. The mathematical simplicity of this method is helpful for system diagnosis, and it also enables real-time computation of MR [13]. The temporal accuracy of this approach greatly depends on the derivative terms, and also depends on the estimation of the chamber volume, as well as the accuracy of the gas analyzers. Based on the method proposed by [13], the delay in response comes from the step size of the derivative term. However, the cost of shortening the derivative step is the noise introduced to the finite difference computation [16]. [17] reduced this noise in the finite difference computation with a central difference method and wavelet noise reduction, and the derivative step size can be shortened to three minutes, effectively reducing the time lag to two minutes in comparison with ground truth MR signals.

In this paper, we base our work on the third school approach, and test the feasibility of adopting the shortest possible step size for the derivative term, in order to maximize the temporal resolution. Unlike the first and second school approaches, there is no system characterization method (e.g. transfer function of the system is known) built into this approach. Therefore, careful step-by-step corrections are required prior to studies to obtain accurate measurements. For example, gas analyzer drift needs to be monitored on a daily basis and regularly calibrated; the offset between gas analyzers monitoring input air and output air also needs to be determined and compensated for; the chamber volume needs to be re-assessed every time the room furniture setting is changed. Even with these maintenance steps, errors can still stem from the unstable incoming air source, noise introduced by the finite difference term in the calculation, and the reduction in temporal accuracy when recovering high-frequency metabolic signals. These issues are discussed in detail in Section 4. The rest of this section describes the third school approaches on which our work is based.

### 3.1 Calculations of MR

The chamber system estimates *VO*_2_ (unit: l/min) and *VCO*_2_ (unit: l/min) by measuring the changes in O_2_ (range: 0.20–0.21) and CO_2_ concentrations (range: 0–0.01) in the entire room. The ways to derive *VO*_2_ and *VCO*_2_ from gas concentrations are captured in Eq 1, using the methods detailed in [13, 18]:

$$\begin{array}{c} \left\{ \begin{array}{cc} VO_{2} & =-F\times \left(O_{2}^{Out}\times H-O_{2}^{In}\right)-V\times {\dot{O_{2}}}^{Out}\\ VCO_{2} & =F\times \left(CO_{2}^{Out}\times H-CO_{2}^{In}\right)+V\times {\dot{CO_{2}}}^{Out} \end{array}\right. \end{array}$$ (1)

In Eq 1, F is the flow rate of the fresh medical air going into the chamber; $O_{2}^{In}$, $CO_{2}^{In}$, $O_{2}^{Out}$, and $CO_{2}^{Out}$ are the O_2_ and CO_2_ levels in the incoming air and chamber air, respectively; ${\dot{O_{2}}}^{Out}$ and ${\dot{CO_{2}}}^{Out}$ are the time derivatives of the O_2_ and CO_2_ concentration levels in the chamber air. These derivative terms in discrete form are:

$$\begin{array}{c} \left\{ \begin{array}{cc} {\dot{O_{2}}}^{Out}\left[n\right] & =\frac{{O_{2}}^{Out}\left[n\right]-{O_{2}}^{Out}\left[n-k\right]}{k}\\ {\dot{CO_{2}}}^{Out}\left[n\right] & =\frac{{CO_{2}}^{Out}\left[n\right]-{CO_{2}}^{Out}\left[n-k\right]}{k} \end{array}\right. \end{array}$$ (2)

where n is the time index of the discrete signals, and k is the step size of the backward derivative term. *V* is the volume of the chamber, which is determined prior to validation tests, using the methods detailed in Section 3.2. H is the Haldane coefficient [13]:

$$\begin{array}{c} \left\{ \begin{array}{c} N_{2}^{In}=1-\left(O_{2}^{In}+CO_{2}^{In}\right)\\ N_{2}^{Out}=1-\left(O_{2}^{Out}+CO_{2}^{Out}\right)\\ H=\frac{N_{2}^{In}-\dot{N_{2}^{Out}}\times V/F}{N_{2}^{Out}} \end{array}\right. \end{array}$$ (3)

Lastly, given *VO*_2_ and *VCO*_2_, EE (unit: kcal, in our paper, we follow the convention of using “small calorie”, kcal is thus equivalent to one Calorie, or “big calorie”, 1 kcal = 4184 J), or MR (unit: kcal/min) can be derived using Weir’s equation, ignoring the urinary nitrogen term [4]:

$$\begin{array}{c} MR=3.941\times VO_{2}+1.106\times VCO_{2} \end{array}$$ (4)

### 3.2 Determining chamber volume

Although one can roughly estimate the chamber volume by measuring the dimensions of the chamber and the equipment in the chamber, the estimation error can be large due to the irregular shapes of the furniture and the room layout. Common practice for determining the chamber volume is through a “washout” test, in which fresh air is sent into an empty chamber which has a higher CO_2_ concentration after a human study. The CO_2_ concentration signal in the empty chamber is then captured until it approaches the CO_2_ level in the fresh air. This signal represents the zero-input response of the chamber system. Therefore, the chamber volume as a parameter of Eq 1 can be determined by solving a series of zero-input differential equations. The zero-input response equation can be derived from Eq 1:

$$\begin{array}{c} \frac{V}{F}{\dot{CO_{2}}}^{Out}+{CO_{2}}^{Out}-{CO_{2}}^{In}=0 \end{array}$$ (5)

where ${CO_{2}}^{In}$ is known. The discrete form of the general solution to this zero-input response differential equation is [19]:

$$\begin{array}{c} CO_{2}^{Out}\left[n\right]=A\times e^{-\frac{F[n]}{V}}+C; \end{array}$$ (6)

**Fig 3:**
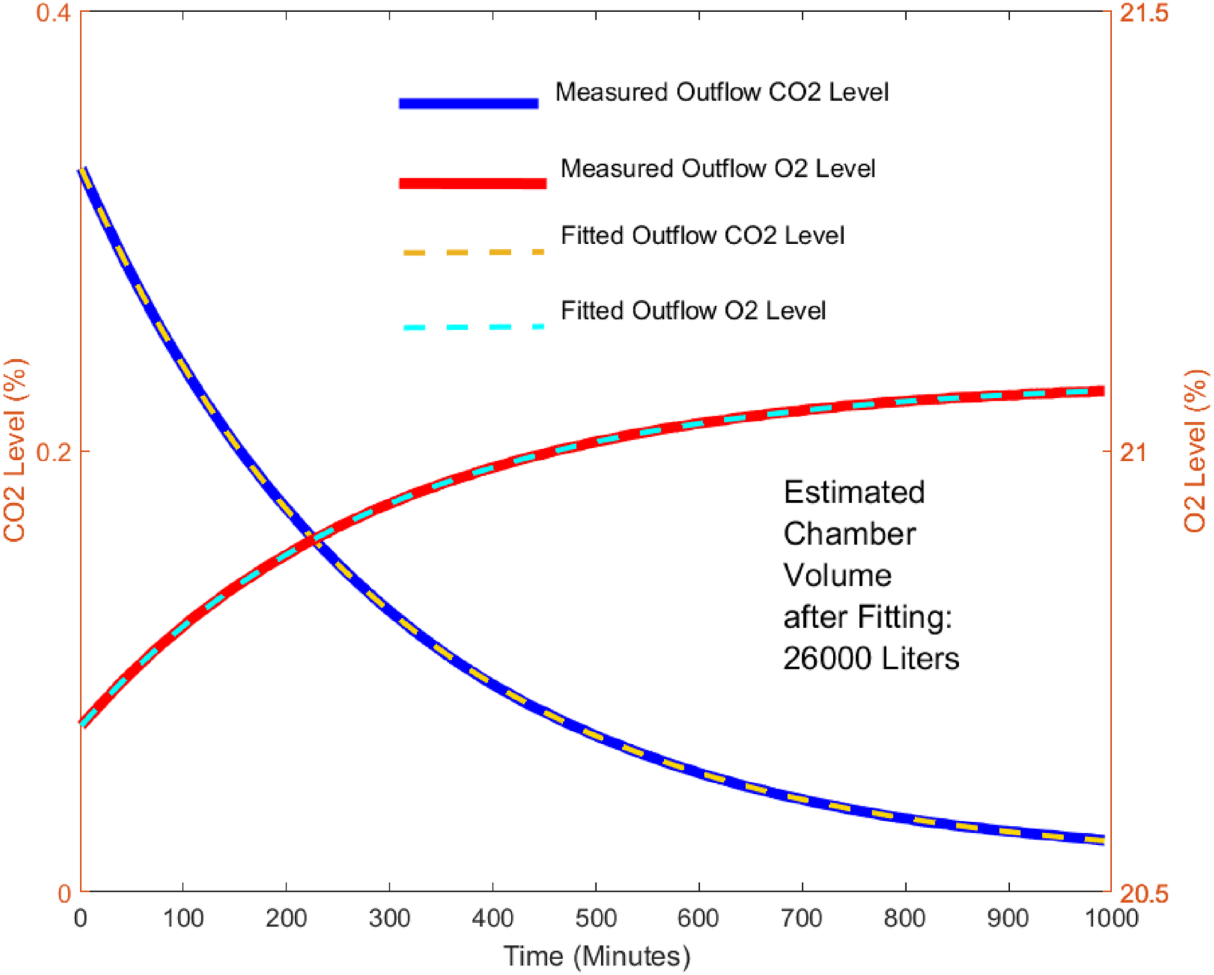
Exponential curve fitting for chamber volume estimation. Exponential curves were fitted into measured O_2_ and CO_2_ gas concentrations from a washout test. CO_2_ and O_2_ estimated volumes should be similar in a properly functioning and calibrated system.

By recording $CO_{2}^{Out}$ and *F* over an extended period, the coefficient A, offset C, and the chamber volume V can be found by fitting an exponential curve. Similarly, this method can be applied by recording the $O_{2}^{Out}$ curve as well. Fig 3 shows the recorded $O_{2}^{Out}$ and $CO_{2}^{Out}$ curves over 16 hours for the big chamber under the zero-input condition, and the fitted curves after finding A, C, and V in Eq 6. The big chamber volume, V, was then found to be 26000 Liters.

### 3.3 Calibration

Due to the drift in gas analyzers, it is necessary to calibrate the gas analyzer prior to human subject studies. Reference points for calibration can be obtained by mixing gases onsite to known concentration levels. The gas mixing is done using a gas blender which is comprised of MFCs and developed by MEI Research. Each MFC is pre-validated by a primary flow standard (Mesa Labs, ML-800). During the calibration stage, three MFCs will be used to regulate the flow rates of N_2_, O_2_, and CO_2_ gases. These three gases then flow into a manifold that mixes them at a known combination of flow rates. The flow rates are pre-determined to generate O_2_ levels from 20% ∼ 21%, and CO_2_ levels from 0% ∼ 1%. The reference points of O_2_ and CO_2_ concentrations can be calculated using Eq 7.

$$\begin{array}{c} \left\{ \begin{array}{cc} O_{2}\% & =\frac{MFC_{O_{2}}}{MFC_{O_{2}}+MFC_{CO_{2}}+MFC_{N_{2}}}\times 100\%\\ CO_{2}\% & =\frac{MFC_{CO_{2}}}{MFC_{O_{2}}+MFC_{CO_{2}}+MFC_{N_{2}}}\times 100\% \end{array}\right. \end{array}$$ (7)

where $MFC_{O_{2}}$, $MFC_{CO_{2}}$, and $MFC_{N_{2}}$ are the flow rates (units: l/min) of O_2_, CO_2_, and N_2_ being mixed in the blender respectively. The results of the calibration are shown in the supporting information S1 File: **Calibration Results**, **Figs A-B**.

### 3.4 Infusion validation

For validation, the ground truth input and output of the system are obtained by infusing N_2_ and CO_2_ into an empty chamber with known flow rates controlled by the MFCs, mimicking the effect of the consumption of O_2_ and production of CO_2_ during human respiration. Thus, the ground truth input of the system can be found by:

$$\begin{array}{c} \left\{ \begin{array}{cc} VO_{2}^{Exp} & =\frac{F\times \;O_{2}^{In}\times MFC_{N_{2}}}{MFC_{N_{2}}+F\times N_{2}^{In}}\\ VCO_{2}^{Exp} & =\frac{F\times (MFC_{CO_{2}}\times N_{2}^{In}-MFC_{N_{2}}\times CO_{2}^{In})}{MFC_{N_{2}}+F\times N_{2}^{In}} \end{array}\right. \end{array}$$ (8)

where $N_{2}^{In}$ is the estimated N_2_ concentration in the inflow air, $MFC_{CO_{2}}$ and $MFC_{N_{2}}$ are the infusion flow rates of CO_2_ and N_2_ respectively; $VO_{2}^{Exp}$, $VCO_{2}^{Exp}$ (units: l/min) are the expected values of *VO*_2_ and *VCO*_2_ respectively; the expected MR values *MR*^*Exp*^ (unit: kcal/min) can be found using Weir’s equation. To obtain the ground truth output of the system, a recursive method to transform $VO_{2}^{Exp}$ to $O_{2}^{OutExp}$ was adapted from [17] by rearranging Eq 1 and substituting from Eq 2:

$$\begin{array}{c} \left\{ \begin{array}{cc} O_{2}^{OutExp}\left[n\right] & =O_{2}^{Out}\left[n\right],\;\text{for}\;n=1;\\ O_{2}^{OutExp}\left[n\right] & =\frac{1}{V+F[n]\times H[n]}\times (-VO_{2}^{Exp}\left[n\right]\\ & \;+\;F\left[n\right]\times O_{2}^{In}\left[n\right]+V\times O_{2}^{Out}\left[n-1\right]),\;\text{for}\;n>1 \end{array}\right. \end{array}$$ (9)

where $O_{2}^{OutExp}$ is the expected concentration level of $O_{2}^{Out}$, and step size *k* is chosen to be 1. Similarly, $CO_{2}^{OutExp}$ can also be obtained using this recursive method.

### 3.5 Run-time MR signals

**Fig 4:**
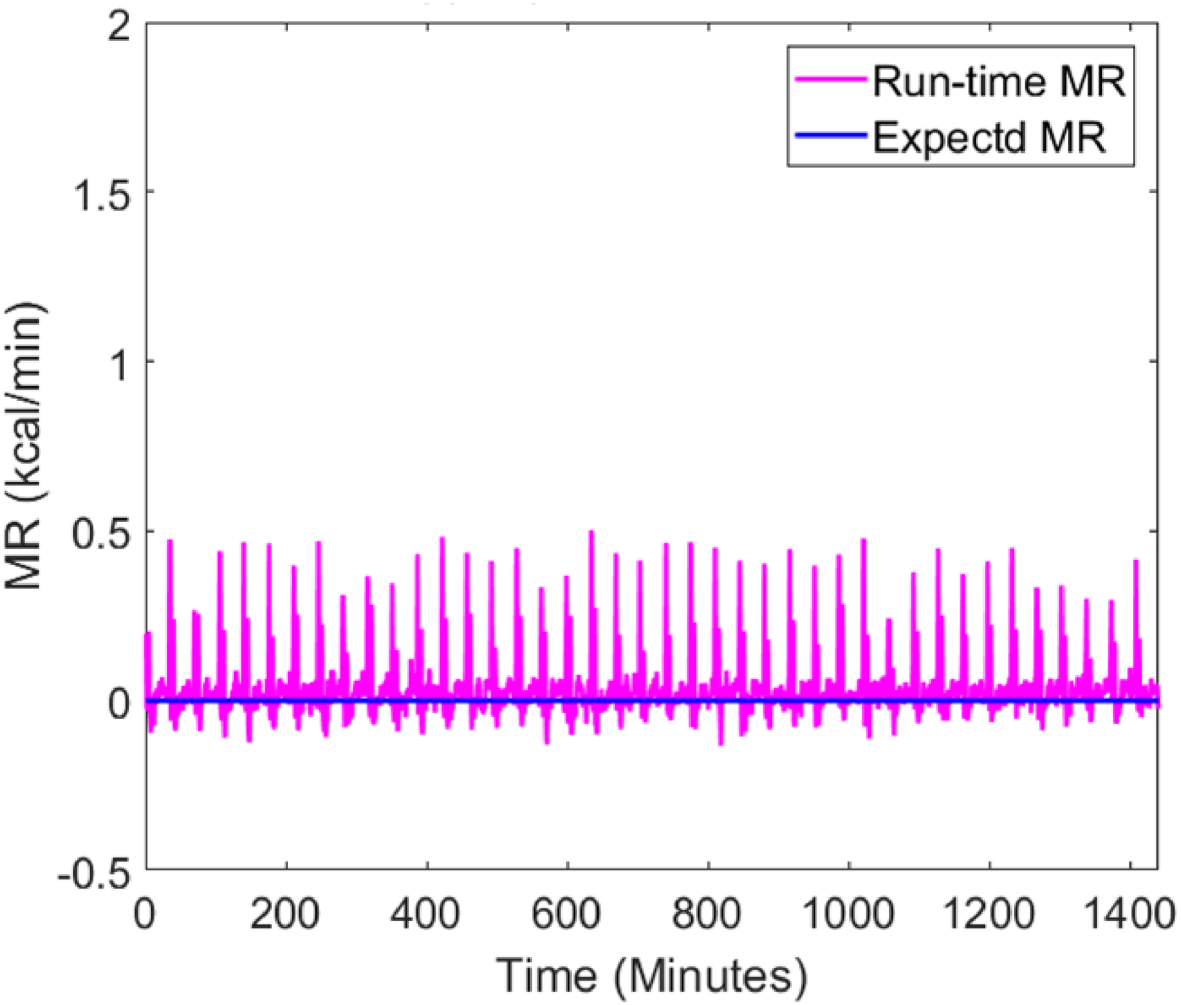
Baseline MR (kcal/min) measured in an empty chamber. The expected MR (solid blue) signal for an empty chamber is constant zero, and the run-time MR (solid magenta) shows there is spiky noise in the measured MR.

**Fig 5:**
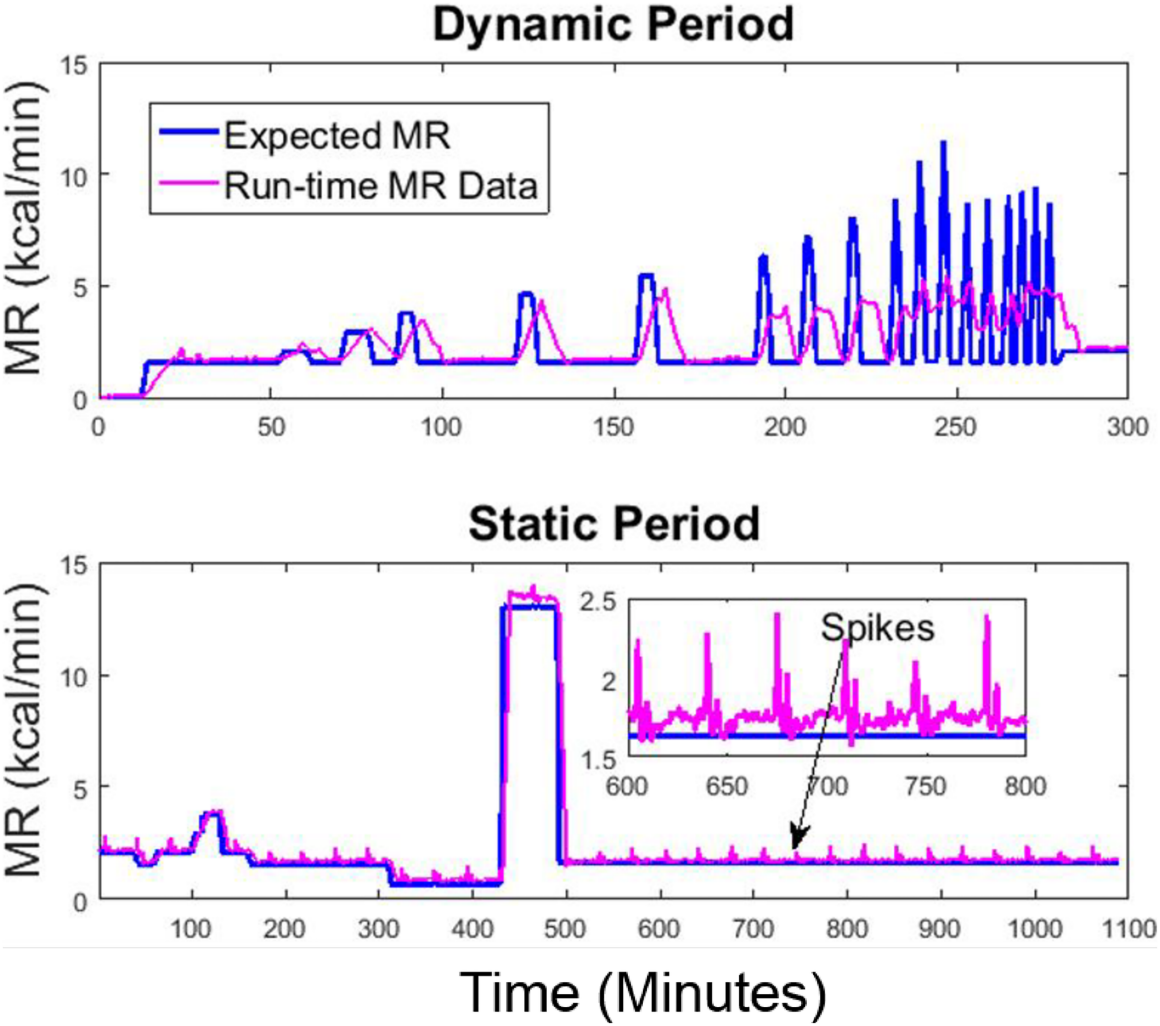
MR measured during a gas infusion test session in the big chamber. The run-time MR signal is compared to the expected MR signal for a dynamic period (top) simulating MR during physical activities and a static period (bottom) simulating MR during sedentary periods and sleep.

The run-time MR signals during an empty chamber test and an infusion test are shown in Figs 4 and 5. In the empty chamber test, inflow air was sent into the chamber at 60 l/min as normal but the chamber had no subject in it. In this setting, the expected MR values should be constant zero (baseline of the system), regardless of the inflow rates and the volume of the chamber.

The infusion session lasted for 24 hours, including a dynamic period to simulate high-frequency metabolic signals during physical activities and a static period to simulate low-frequency metabolic signals during sedentary activities and sleep. In the dynamic period, the high-frequency metabolic signals were simulated using 8-minute, 5-minute, 3-minute, and 1-minute step input functions, with 3-minute or 5-minute resting intervals in between. The raw signals were computed using Eq 1 with an 8-minute backward derivative term, implemented in real-time in LabVIEW. Owing to the accurate estimation of chamber volume and gas analyzer calibration, the raw signals measure the longer term resting MR accurately. However, when the MR signals are more dynamic (e.g. 1-minute high-level metabolic activities with only 3-minute intervals in between), the temporal resolution is lost in the raw signals during the dynamic period.

Figs 4 and 5 also reveal the presence of artifact spikes in the raw MR signals. Based on our observations over half a year, we identified that the error was caused by the varying O_2_ level in the medical air. The CO_2_ level also varies in the medical air, but due to its negligible magnitude, we focused on removing the O_2_ spikes using a pre-processing algorithm. The reason and remedy for the spikes are detailed in the supporting information S2 File: **Spike Removal Method**, **Figs A-D**.

### 3.6 Human exercise data

We also collected data on one healthy female subject (age: 38, weight: 59kg, BMI: 22) during treadmill exercise. The study protocol was approved by the Virginia Commonwealth University’s IRB and the written consent was obtained before the study. During the study, the subject entered the chamber and rested for 25 minutes in order for the chamber to equilibrate as well as to obtain the baseline of the subject, and then was cued to walk on the treadmill at three different speeds (3.4 miles/hour, 3 miles/hour and 5.2 miles/hour) for about 15 minutes and to jog for a short time. The timestamps of the start and end of each bout of exercise were annotated in the software recording the MR.

## 4 Proposed method

In this section, we present a noise-reduction method to reduce the noise created by 1-minute derivative finite estimation to best improve the temporal accuracy. Besides the low pass filter characteristic of the chamber system [6] that reduces its temporal accuracy, the accuracy may be further decreased by finite difference calculations. The noise reduction process for derivative terms usually involves numeric finite difference estimation methods (such as Newton forward difference or weighted finite difference) combined with filtering techniques, such as central difference methods. However, these methods usually require a relatively long step size to smooth out the noise in the numeric derivatives, hindering the recovery of MR signals with higher frequency. Therefore, in the proposed method, we use a 1-minute backward difference calculation, which provides the highest time resolution given a sampling rate of once per minute. The numeric noise caused by this short-term derivative estimation will be compensated for by other filtering techniques. To preserve the transitional edges in the signal, we adopted a total variation denoising (TVD) technique. In comparison to Tikhonov regularization, this *ℓ*_1_-norm regularization technique is superior in preserving edges without over-penalizing the discontinuity of the signal, thus it does not have the edge smoothing effect of Tikhonov regularization [20]. The TVD method minimizes the difference between a noisy signal *y*[*n*] and a true signal *x*[*n*] while trying to preserve the edge of the noisy signal. The noisy signal *y*[*n*] can be considered as the true signal *x*[*n*] with additive white noise *w*[*n*]:

$$\begin{array}{c} y[n]=x[n]+w[n],n=1,2,3\ldots N \end{array}$$ (10)

The total variation, which measures the fluctuation of a signal *x*[*n*], is defined as the *ℓ*_1_-norm of the derivative of the signal:

$$\begin{array}{c} TV=\sum_{n=1}^{N-1}\left|x\left[n+1\right]-x\left[n\right]\right|\; \end{array}$$ (11)

The goal of the TVD algorithm is to find an approximation to the true signal *x*[*n*] with reduced TV. The closeness to *x*[*n*] is measured by the sum of squared errors:

$$\begin{array}{c} \frac{1}{2}\sum_{n=1}^{N}{\left|y\left[n\right]-x\left[n\right]\right|}^{2} \end{array}$$ (12)

Thus the TVD problem can be seen as minimizing the sum of squared errors between *x*[*n*] and *y*[*n*] penalized by the TV of *x*[*n*]:

$$\begin{array}{c} \underset{x\in R^{N}}{arg\;min}\;\{f\left(x\right)=\frac{1}{2}\sum_{n=1}^{N}{\left|y\left[n\right]-x\left[n\right]\right|}^{2}+\lambda \sum_{n=1}^{N-1}\left|x\left[n+1\right]-x\left[n\right]|\right\} \end{array}$$ (13)

where *λ* is a positive regularization parameter. By tuning *λ*, the reduction of TV in the noisy signal can be adjusted. *y*[*n*] is ${\dot{O_{2}}}^{Out}\left[n\right]$ and *x*[*n*] is the true signal of ${\dot{O_{2}}}^{Out}\left[n\right]$. Eq 13 poses a convex but not smooth optimization problem [21] [22] because of the *ℓ*_1_-norm regularization term, which can be difficult to solve. To solve this problem, a Majorization-Minimization (MM) algorithm is adopted to reduce the computational complexity [23] [24]. In this algorithm, a sequence of convex functions, *G*_*s*_(*x*) (where *s* = 0, 1, 2, …), is used to approximate *f*(*x*), such that:

$$\begin{array}{c} G_{s}\left(x\right)\geq f\left(x\right),\forall x \end{array}$$ (14)

$$\begin{array}{c} and\;G_{s}\left(x_{s}\right)=f\left(x_{s}\right) \end{array}$$ (15)

The optimization problem posed by Eq 13 then becomes:

$$\begin{array}{c} x_{s+1}=\underset{x\in R^{N}}{arg\;min}\;G_{s}\left(x\right) \end{array}$$ (16)

and the solution to this optimization problem then becomes:

$$\begin{array}{c} x_{s+1}=y-D^{T}{\left(\frac{1}{\lambda }\Lambda_{s}+DD^{T}\right)}^{-1}Dy \end{array}$$ (17)

where ***x*** is initialized first as ***x*** = ***y***. *D* is the differential matrix, and *D*^*T*^ is its transpose:

$$\begin{array}{c} D=[\begin{array}{ccccc} -1 & 1 & 0 & \ldots & 0\\ 0 & -1 & 1 & \ldots & 0\\ \vdots & \ddots & \ddots & \vdots & \vdots \\ 0 & \ldots & -1 & 1 & 0\\ 0 & \ldots & 0 & -1 & 1 \end{array}] \end{array}$$ (18)

and Λ_*s*_ is the diagonal matrix of difference vectors *D* ***x***_*s*_:

$$\begin{array}{c} \Lambda =[\begin{array}{cccc} |x_{2}-x_{1}| & \\ & |x_{3}-x_{2}| & \\ & \ddots & \\ & & |x_{k+1}-x_{k}| & \\ & & \ddots \\ & & & |x_{N}-x_{N-1}| \end{array}] \end{array}$$ (19)

The computational algorithm for this solution is implemented in Matlab as described in [24]. The regularization parameter *λ* is empirically determined by comparing ${\dot{O_{2}}}^{OutExp}$ with measured ${\dot{O_{2}}}^{Out}$, and the *λ* that provides the minimum root mean square error (RMSE) between ${\dot{O_{2}}}^{OutExp}$ and ${\dot{O_{2}}}^{Out}$ is used.

## 5 Results and discussion

**Fig 6:**
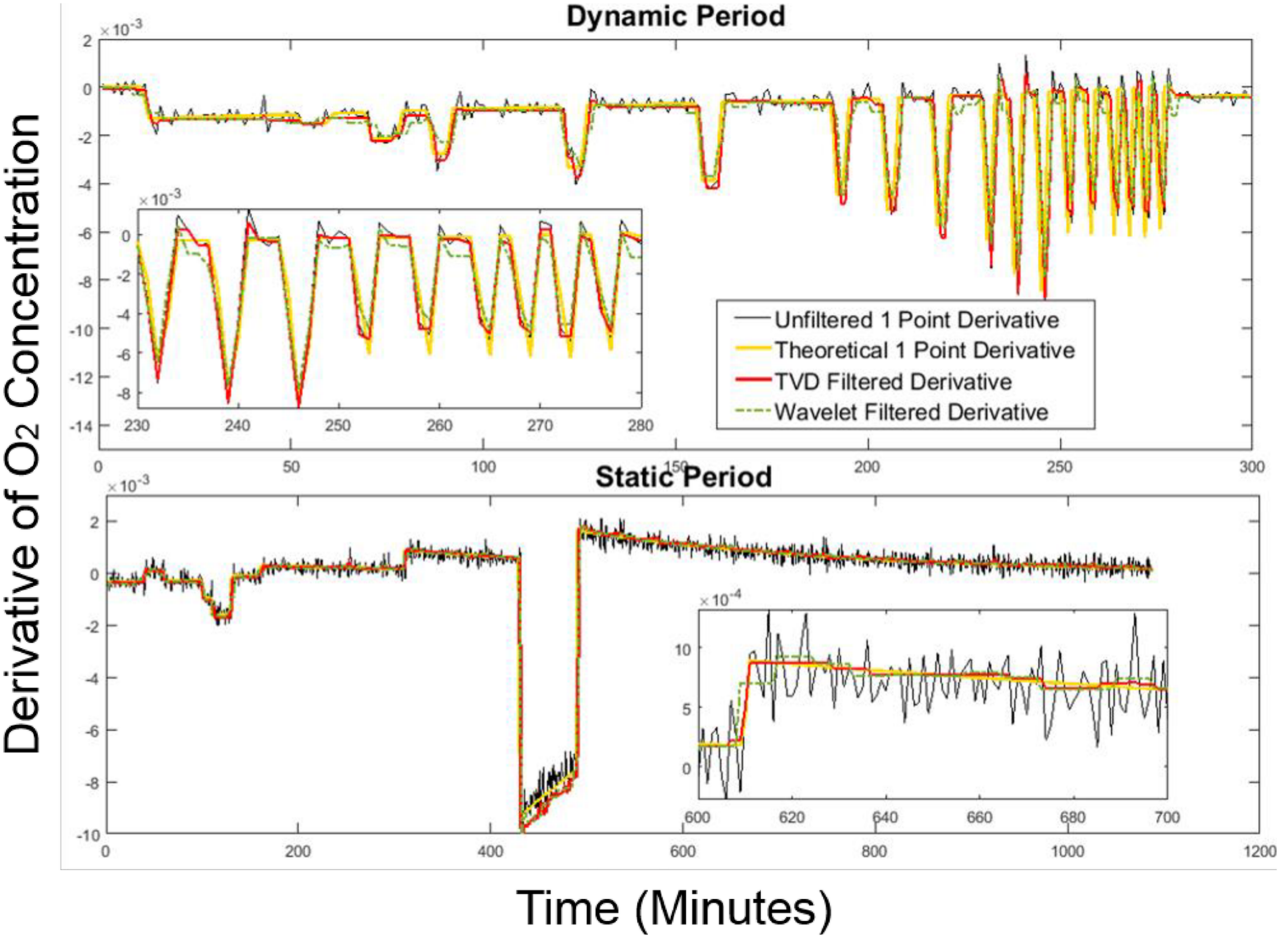
1-minute backward derivative signals filtered by TVD and Wavelet. Both methods were compared against the theoretical derivative data in a 24-hour infusion session.

22 infusion sessions were run to assess chamber performance. To evaluate the noise reduction technique’s performance, we also compared the TVD filtering method with three other commonly used noise reduction methods: an 8-point moving average filter, a 6-minute locally weighted scatterplot smoothing (Lowess) regression method, and a Wavelet correction method used in [17]. The results are presented below. The results of the 1-minute backward derivative signal after noise reduction are shown in Fig 6. The theoretical derivative was obtained by taking the 1-minute backward derivative terms of $O_{2}^{OutExp}$ computed using Eq 9.

**Fig 7:**
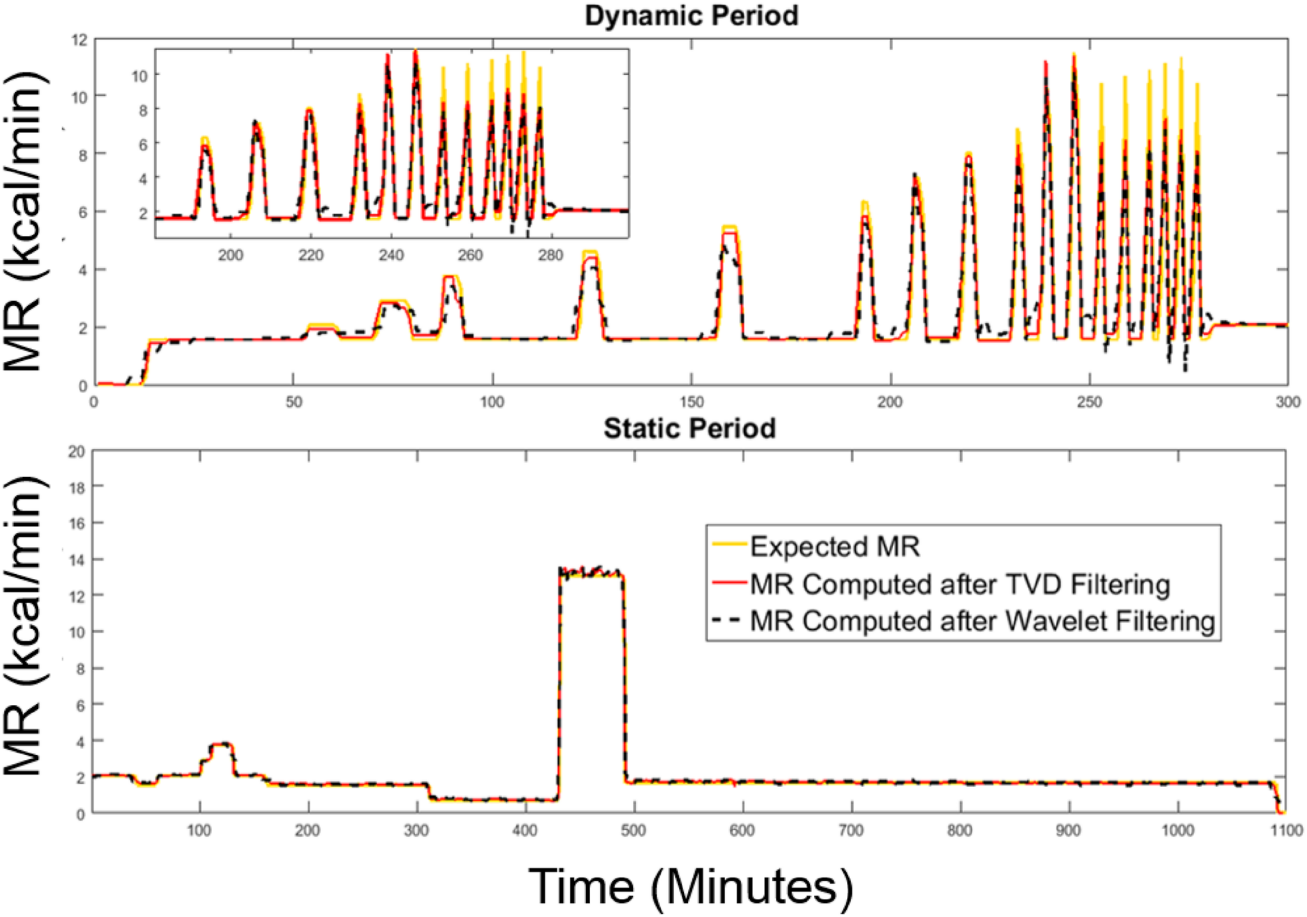
MR signals recovered by TVD and Wavelet. Both methods were compared against expected MR for the dynamic period (top) and the static period (bottom) in a 24-hour infusion session.

**Fig 8:**
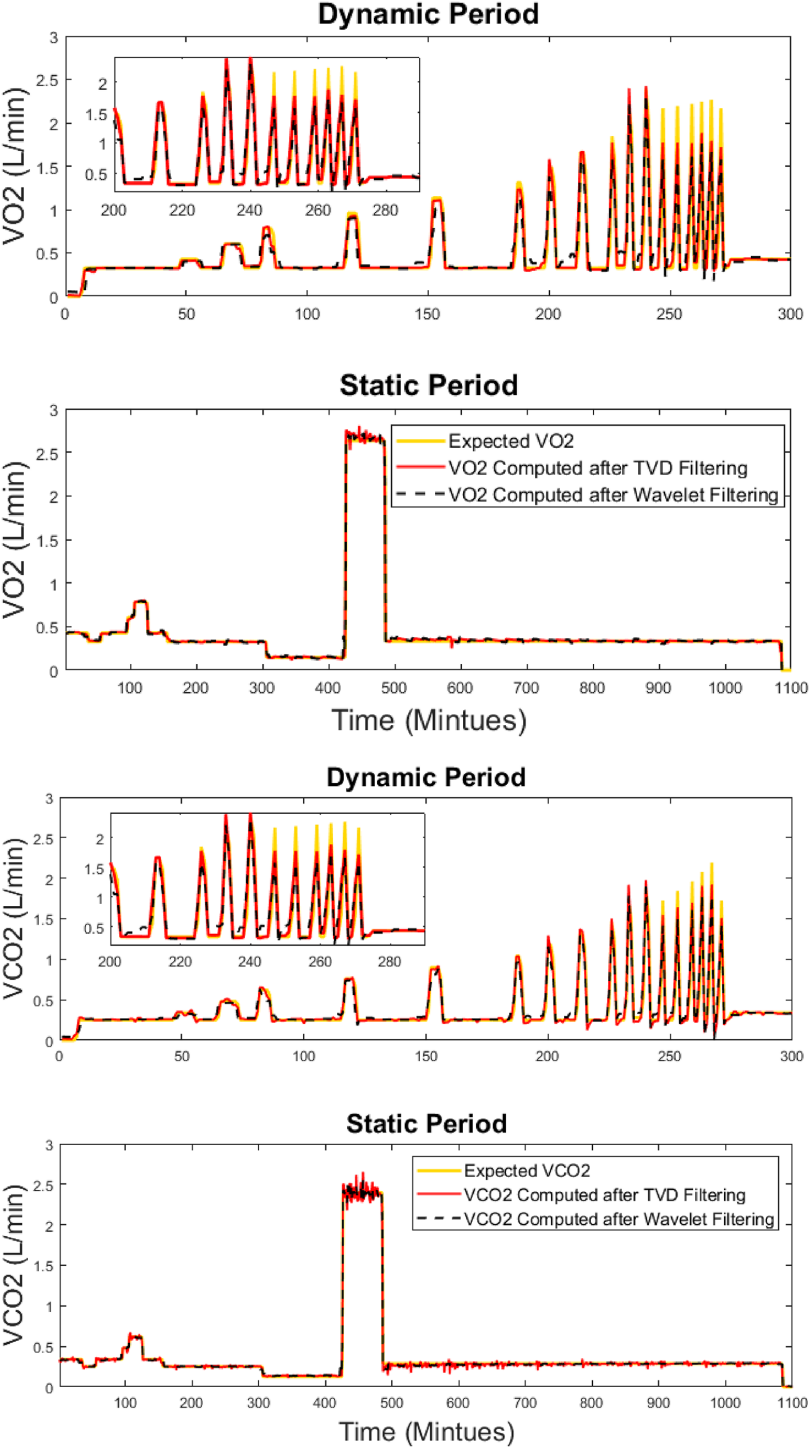
Gas exchange signals (*VO*_2_ and *VCO*_2_) recovered by TVD and Wavelet. Both methods were compared against expected levels for the dynamic period (top) and the static period (bottom) in a 24-hour infusion session.

As shown in Figs 7 and 8, the proposed method recovers the simulated MR signals as well as the signals of gas exchange rates with no delay in the step response. This is suitable for evaluating exercise studies in which the time to reach a steady state is an important parameter in evaluating the human subject’s physique.

**Fig 9:**
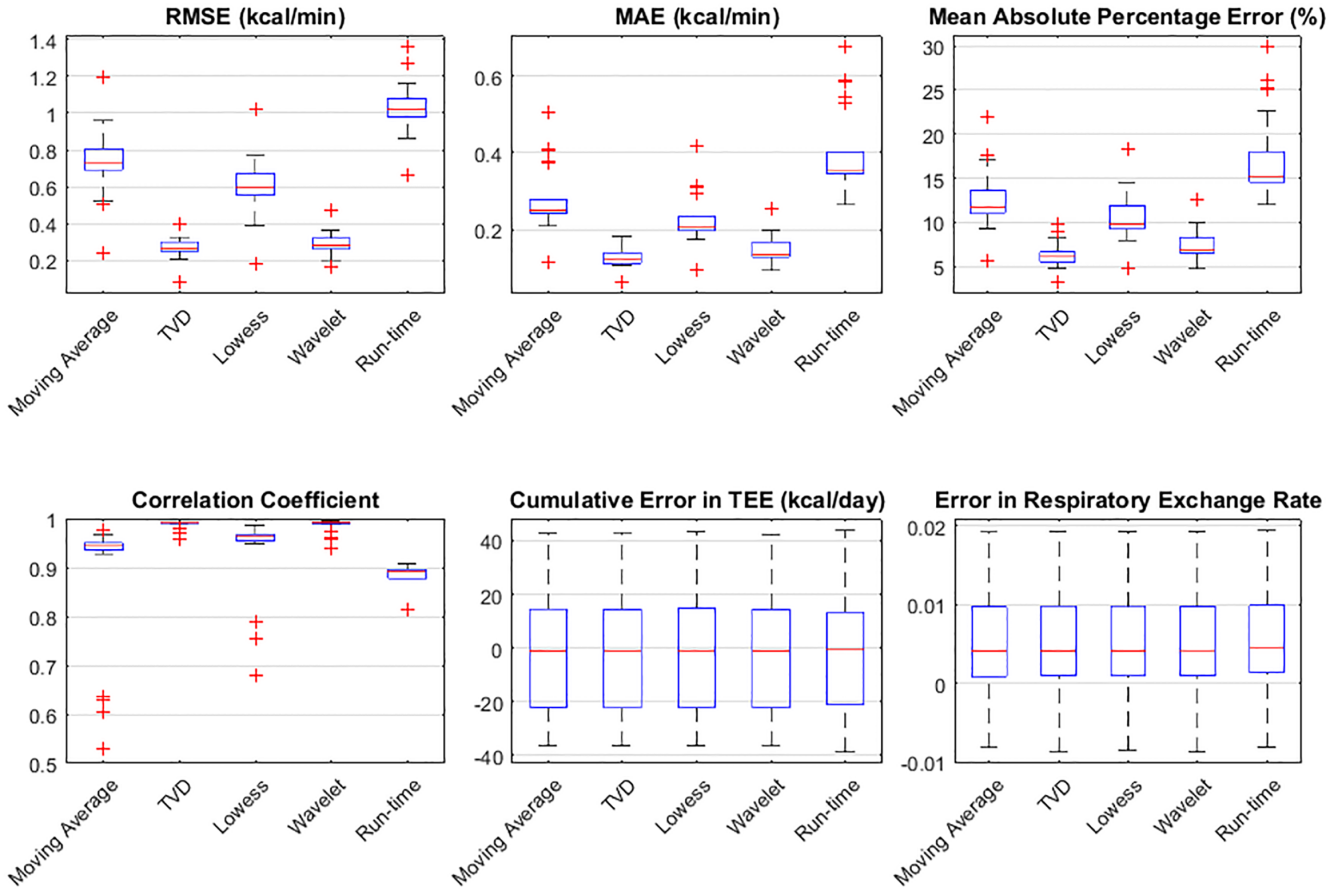
Error metrics used to evaluate the performance of the filter methods. Error metrics include RMSE, MAE, MAPE, correlation coefficient, cumulative error in TEE per day, and absolute error in respiratory exchange ratio per day. Measured MR is compared to the expected MR obtained during infusion validation.

Six error metrics—RMSE, mean absolute error (MAE), mean absolute percentage error (MAPE), correlation coefficient, cumulative error in total EE (TEE) per day, and absolute error in respiratory exchange ratio (RER) per day, are used to evaluate the performance of the proposed system by comparing the measured MR against the expected MR obtained during infusion validation. The results are presented in Fig 9 based on data from 22 infusion sessions. Among the six error metrics, RMSE evaluates the measured MR with a severe penalty on the outliers, MAE evaluates the measured MR without penalizing the outliers, MAPE is a standard error metric to evaluate the accuracy of a system, and the cumulative error in TEE is computed by summing the processed MR data using the trapezoid integration rule on the 24-hour infusion MR signals. Fig 9 shows that the proposed method best estimates dynamic MR signals evaluated by RMSE, MAE and MAPE. Overall, all methods perform similarly well in terms of the cumulative error in TEE per day (less than 45 kcal/day) and daily RER (absolute error of 0.0051). However, on average, the TVD method shows the lowest error when temporal resolution is considered, with an RMSE of 0.27 kcal/min (18.8 J/s), an MAE of less than 0.13 kcal/min (9 J/s), an MAPE of 6.3%, and the highest correlation coefficient (0.99).

**Fig 10:**
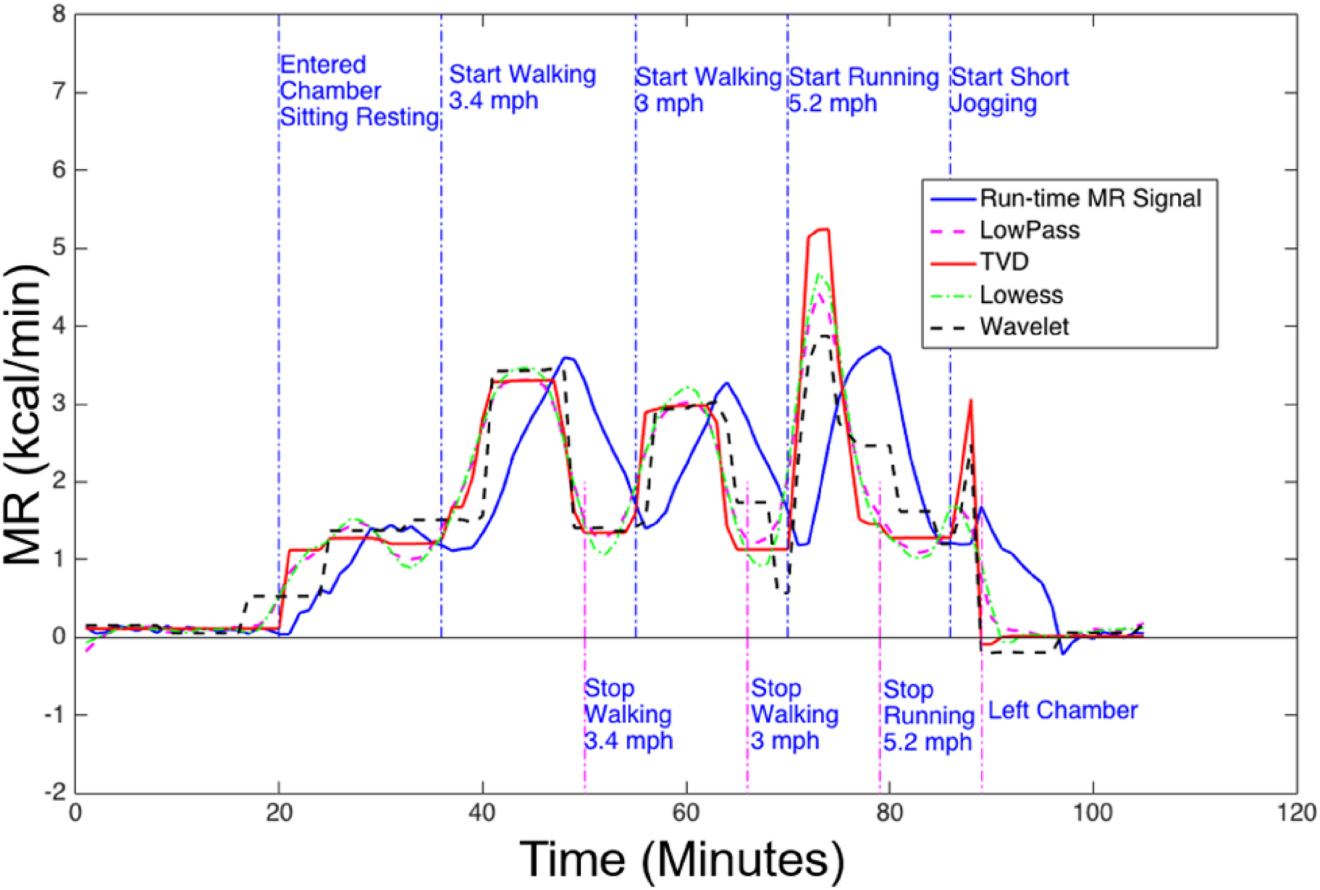
MR signals of a human subject exercising on a treadmill. MR signals were generated during run-time using the 8-minute backward derivative term (solid blue), or computed with the 1-minute backward derivative and then filtered by low-pass (dashed magenta), TVD (solid red), Lowess(dashed green), and Wavelet (dashed black) filters. Adopting the 1-minute derivative term and applying the TVD filtering produced the least delay (best temporal resolution) between the transition edge and the annotated timestamps.

In Fig 10, we demonstrate that in one human subject exercise session, the proposed method recovers the MR signals with sufficient temporal resolution and provides clear transition edges and the flattest estimation in the steady states during exercise. The moving average method and the Lowess method both over-smooth the MR signals, and do not recover the steady state well. Although the Wavelet method is equally good at preserving the edges of the signal during denoising, it can also create artifacts in the signals. Overall, the proposed method estimated such temporal features accurately, by recovering the clear transition edges of MR signals during exercise.

## 6 Conclusion

This paper proposed methods for improving the temporal accuracy in post-processing for human metabolic chambers. Adopting a 1-minute backward derivative term and robust total variation noise reduction techniques, the proposed methods enable the chamber system to capture high-frequency metabolic signals with improved temporal accuracy. This improved performance will allow researchers to incorporate exercise into metabolic chamber study protocols, where the dynamic patterns of metabolic rate signals are of interest.

## Supporting information

S1 File Calibration results. A. Linearity of blender calibrated gas analyzers. B. Bland-Altman analysis for blender calibration. Red circles are difference between true values and using un-calibrated gas analyzer readings and blue diamonds are Bland-Altman plots using calibrated gas analyzer readings.
(PDF)

S2 File Spike removal method. A. Unstable gas concentration in medical air. B. Probability plot of the O_2_ signals. C. Post-processing method to remove spikes of O_2_ in incoming air. D. The baseline of a week-long incoming O_2_ signal is preserved after filtering.
(PDF)
