# Supplementary material for: Improving temporal accuracy of human metabolic chambers for dynamic metabolic studies
Source: PLoS One. 2018 Apr 24;13(4):e0193467. doi: 10.1371/journal.pone.0193467 (PMC5916490; doi:10.1371/journal.pone.0193467)
Supplement: S1 File — A. Linearity of blender calibrated gas analyzers. B. Bland-Altman analysis for blender calibration. Red circles are difference between true values and using un-calibrated gas analyzer readings and blue diamonds are Bland-Altman plots using calibrated gas analyzer readings. (PDF) [file pone.0193467.s001.pdf]

S1: Calibration Results

The calibration results are shown in Fig A and Fig B. Both O_2 analyzers and CO_2 analyzers demonstrate good linearity using blender calibration, as shown in Fig A. The agreement between the calibration reference points and the analyzer readings also improved after calibration, as shown on the Bland-Altman plot in Fig B. After calibration, the mean difference (solid green lines) between the readings of the calibrated analyzers and the reference points shifted to the zero lines from the mean difference (solid magenta lines) between the uncalibrated analyzers and the reference points, indicating that the blender calibration corrected the bias and drift in the gas analyzers. The differences between the calibrated analyzers (blue diamond dots) are also within the 95% limits of agreement lines (dotted green lines), indicating negligible random errors of less than 0.007%.

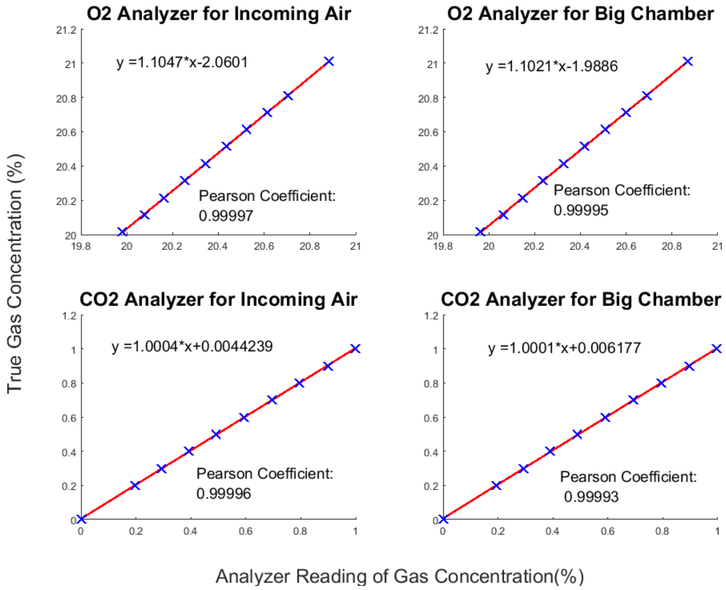

Fig A. Linearity of blender calibrated gas analyzers

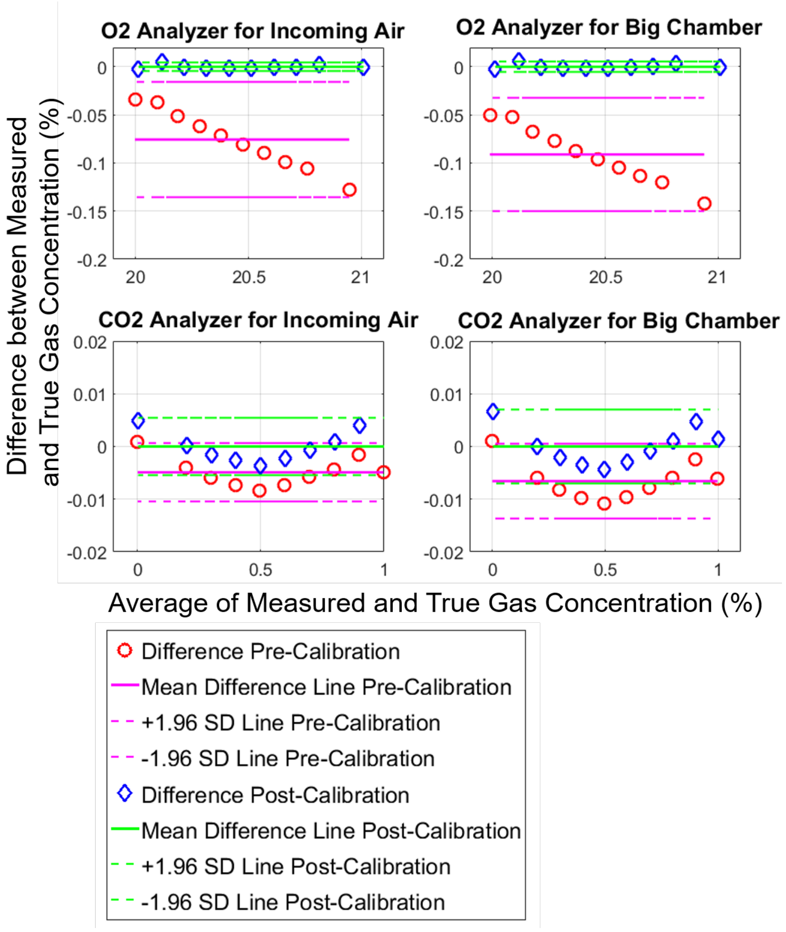

Fig B. Bland-Altman analysis for blender calibration. Red circles are difference between true values and using un-calibrated gas analyzer readings and blue diamonds are Bland-Altman plots using calibrated gas analyzer readings
