# Supplementary material for: Improving temporal accuracy of human metabolic chambers for dynamic metabolic studies
Source: PLoS One. 2018 Apr 24;13(4):e0193467. doi: 10.1371/journal.pone.0193467 (PMC5916490; doi:10.1371/journal.pone.0193467)
Supplement: S2 File — A. Unstable gas concentration in medical air. B. Probability plot of the O2 signals. C. Post-processing method to remove spikes of O2 in incoming air. D. The baseline of a week-long incoming O2 signal is preserved after filtering. (PDF) [file pone.0193467.s002.pdf]

S2 File: Spike Removal Method

In our system, the O_2 level in the medical air (the inflow air) spikes about every 25~35 minutes, and the CO_2 level continuously increases until the O_2 level suddenly increases, as shown in Fig A. Although the small magnitudes of these spikes (about 0.1% in O_2) do not have any significance for the use of medical air in clinical settings (i.e. posing no danger to respiratory functions), because our chambers set out to detect subtle changes in gas concentrations, these spikes do create artifacts in the measured metabolic signals. Since these short-term spikes are diluted once they enter the chamber (due to the chamber's low pass filter behavior), these spikes are not present in the chamber air. This results in spikes being left after the subtraction operation to calculate the difference between O_2^{In} and O_2^{Out} in Equation 1, hence creating artificial spikes in VO_2 and MR estimation.

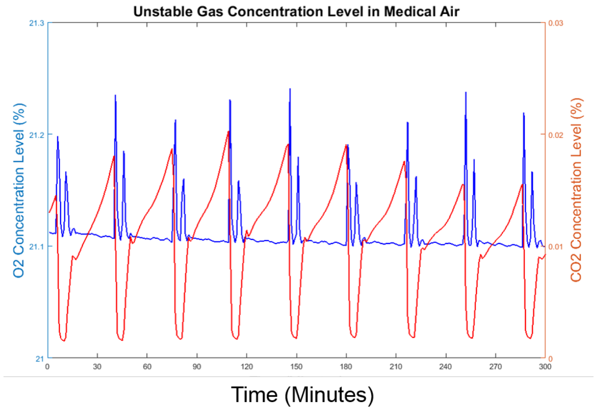

Fig A. Unstable gas concentration in medical air

A fix for this problem could be installing a large gas tank, or a buffer zone (usually equipped in the "pull" type of calorimeter, but not the "push" type) between the medical air source and its inlet to the chamber, thus pre-diluting these spikes in real time. However, since there is a space limitation on placing a large tank (roughly 4500 liters), in this paper we consider a practical solution in post-processing.

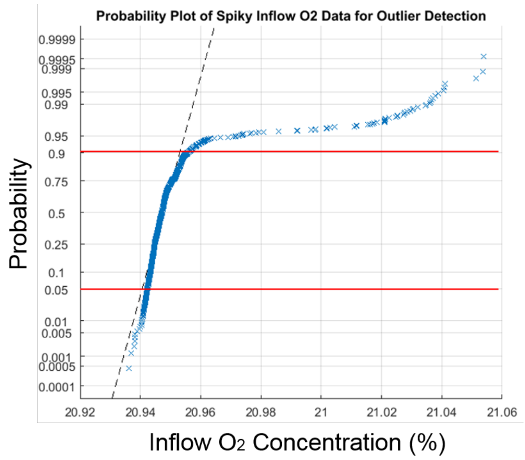

Fig B. Probability plot of the O_2 signals

Since spikes in the signals can be considered as outliers, we designed an outlier removal algorithm that removes data points above the 90% percentile and below the 5% percentile of the

entire distribution. The thresholds 90% and 5% were empirically determined after examining the normal probability plot of the O_2^{In} signals using the Matlab function *probplot*, as shown in Fig B. Comparing with a theoretical normal distribution (i.e. the straight dotted line), above the 90% percentile and below the 5% percentile, the O_2^{In} data deviate from the theoretical normal distribution, and thus can be detected as outliers and removed. After the removal of the outliers, a linear interpolation algorithm was used to impute the removed data, as shown in Fig C.

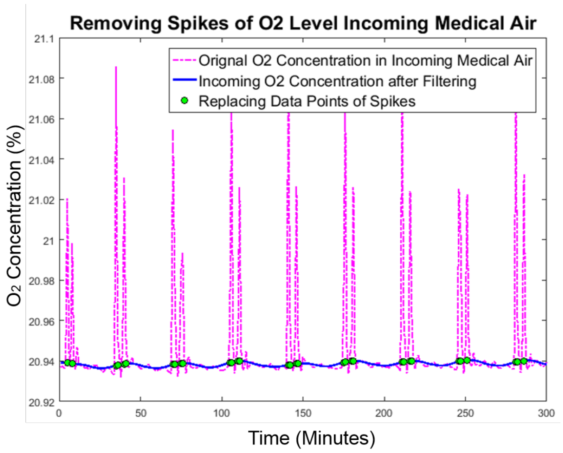

Fig C. Post-processing method to remove spikes of O_2 in incoming air

When no subject is in the chamber, the chamber air concentration will be similar to the baseline medical air concentration and the ambient air concentration, without the spikes. Therefore, to test whether the proposed filtering algorithm recovers the baseline of the Inflow O_2 level, we monitored the chamber air with no subject in it for seven days, as shown in Fig D. The ambient O_2 level changed diurnally during weekdays, with the lowest O_2 level around mid-day and the highest O_2 level around midnight, due to the high traffic flow and dense population around the hospital area where the chamber is located. This effect was apparent during the Independence Day holiday when there was no traffic around, during which the O_2 level increased and stabilized. The O_2 levels in the incoming medical air follow this trend in the ambient air closely after the spikes are removed. The two O_2 levels have a Pearson correlation coefficient of 0.61, and a negligible offset of 0.002%, indicating that the filtering technique preserves the baseline level of the incoming medical air very well.

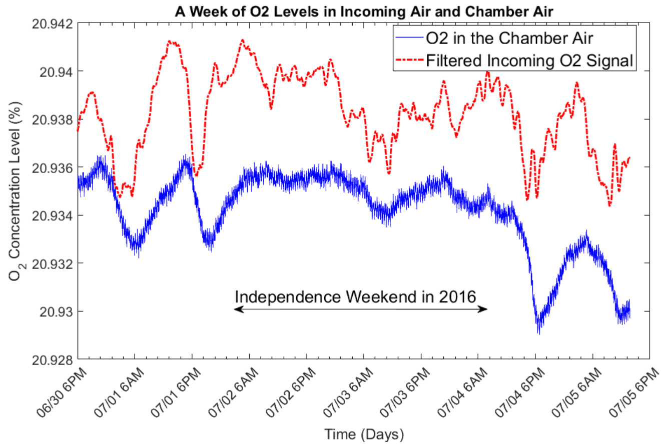

Fig D. The baseline of a week-long incoming O_2 signal is preserved after filtering
